# Supplementary material for: ATP synthase F1 subunits recruited to centromeres by CENP-A are required for male meiosis
Source: Nat Commun. 2018 Jul 13;9:2702. doi: 10.1038/s41467-018-05093-9 (PMC6045659; doi:10.1038/s41467-018-05093-9)
Supplement: Supplementary file 1 — Supplementary Information [file 41467_2018_5093_MOESM1_ESM.pdf]

Supplementary Information

ATP synthase F_1 subunits recruited to centromeres by CENP-A are required for male meiosis.

Collins et al.

Supplementary Figure 1

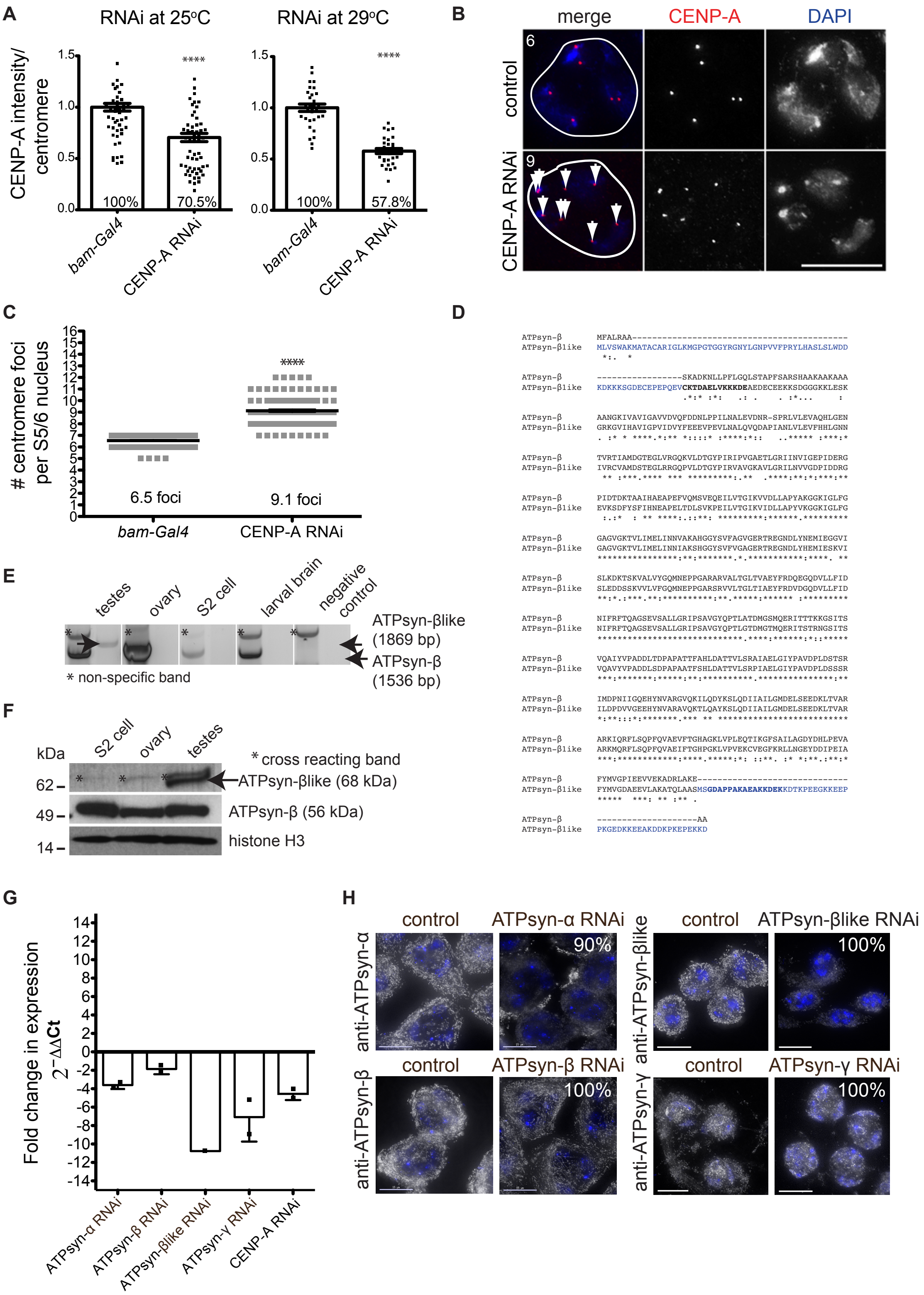

Supplementary Figure 1. Knockdown of CENP-A and ATP synthase F_1 subunits in testis.

A: Centromeric CENP-A fluorescent intensity per nucleus in control (*bam-Gal4*) S5/6 nuclei and nuclei RNAi-depleted of CENP-A at 25°C (n=50 nuclei) or 29°C (n=30 nuclei). Statistical significance was determined using an unpaired student's t-test. **** = p<0.0001. Error bars = SEM.

B: Immuno-fluorescent micrograph of control (*bam-Gal4*) S5/6 nucleus or nucleus RNAi-depleted of CENP-A at 29°C stained with antibodies against CENP-A (red) (n=2). DNA is stained with DAPI (blue). White arrows indicate 9 centromere foci in CENP-A RNAi. Scale bar = 15 µm.

C: Quantitation of centromere foci per control (*bam-Gal4*) S5/6 nucleus and nucleus RNAi-depleted of CENP-A at 29°C (n=100 nuclei). Statistical significance was determined using an unpaired student's t-test.**** = p<0.0001. Error bars = SEM.

D: TCOFFEE alignment of ATPsyn-β and ATPsyn-βlike, showing N- and C-terminal extensions of ATPsyn-βlike (blue). Asterix, semi-colon and stop represent full, strong and weak conservation of amino acid properties respectively. Peptides CKTDAELVKKKDE (amino acid 68-80) and GDAPPAKAEAKKDEK (amino acid 575-587) used to generate anti-ATPsyn-βlike antibodies are marked in bold.

E: Expression of ATPsyn-β (1536 bp) and ATPsyn-βlike (1869 bp) in testes, ovary and larval brain tissues and *Dm* S2 cells by RT-PCR (n=2).

F: Western analysis of ATPsyn-β (56 kDa) and ATPsyn-βlike (68 kDa) expression in testes and ovary total protein extracts (n=2).

G: qPCR validation of ATPsyn-α, -β, -βlike, -γ and CENP-A knockdown in adult testes. Error bars = Mean with SD.

H: Immunoflourescent micrograph of control S5/6 nuclei or nuclei RNAi-depleted for ATPsyn-α, -β, -βlike or -γ at 25°C stained with respective antibodies against ATPsyn-α, -β, -βlike or -γ (grey). DNA is stained with DAPI (blue). Percentage of cells with depleted signals is indicated. Scale bar = 15 µm.

Supplementary Figure 2

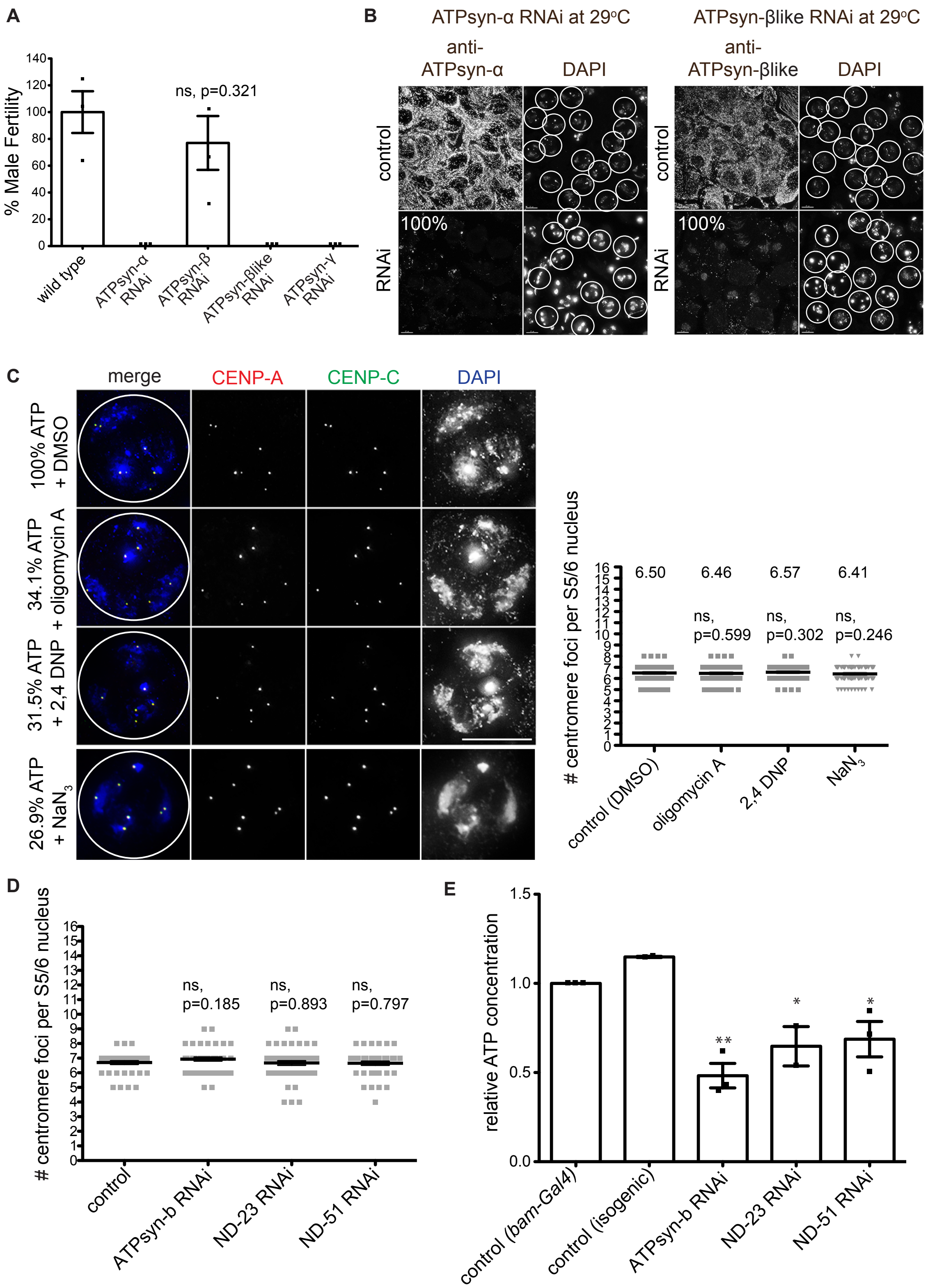

Supplementary Figure 2. Effect of ATP reduction on sister centromere cohesion.
A: Male fertility assay for control adults (TRiP isogenic) or adults in which ATPsyn-α, -β, -βlike and -γ is RNAi-depleted in testes (n=3 individual RNAi experiments). Statistical significance was determined using an unpaired student's t-test. ns = p>0.05. Error bars = SEM.
B: Immuno-fluorescent micrograph of a control 16-cell cyst at 29°C or a cyst RNAi-depleted for ATPsyn-α stained with antibodies against ATPsyn-α (left) or RNAi-depleted for ATPsyn-βlike stained with antibodies against ATPsyn-βlike (right). DNA is stained with DAPI (blue). Nuclei are outlined with white circles. Scale bar = 15 µm.
C: Left:Immuno-fluorescent micrograph of S5/6 nuclei treated with DMSO (control), oligomycin A, 2,4 DNP or NaN_3 stained with antibodies against CENP-A (red) and CENP-C (green). DNA is stained with DAPI (blue). Percentages indicate measured ATP level in adult testes after drug treatments compared to the control (n=3). Scale bar = 10 µm. Right: Quantitation of the number of centromere foci per S5/6 nucleus treated with DMSO (control), oligomycin A, 2,4 DNP or NaN_3 (n=150 nuclei, pooled from three individual experiments). Statistical significance was determined using an unpaired student's t-test. ns = p>0.05. Error bars = SEM.
D: Quantitation of the number of centromere foci per control S5/6 nucleus or nuclei RNAi-depleted for ATPsyn-b, ND-23 or ND-51 (n=1). Statistical significance was determined using an unpaired student's t-test. ns = p>0.05. Error bars = SEM.
E: Relative ATP concentration in control adult testes (isogenic, *bam-Gal4*) or testes RNAi-depleted for CENP-A, ATPsyn-b, ND-23 or ND-51. Experiments were carried out in triplicate and pooled from three individual RNAi experiments. Error bars = SEM. Unpaired t-test compares RNAi knockdowns to the isogenic control. ** = p<0.01, *=p<0.05.

Supplementary Figure 3

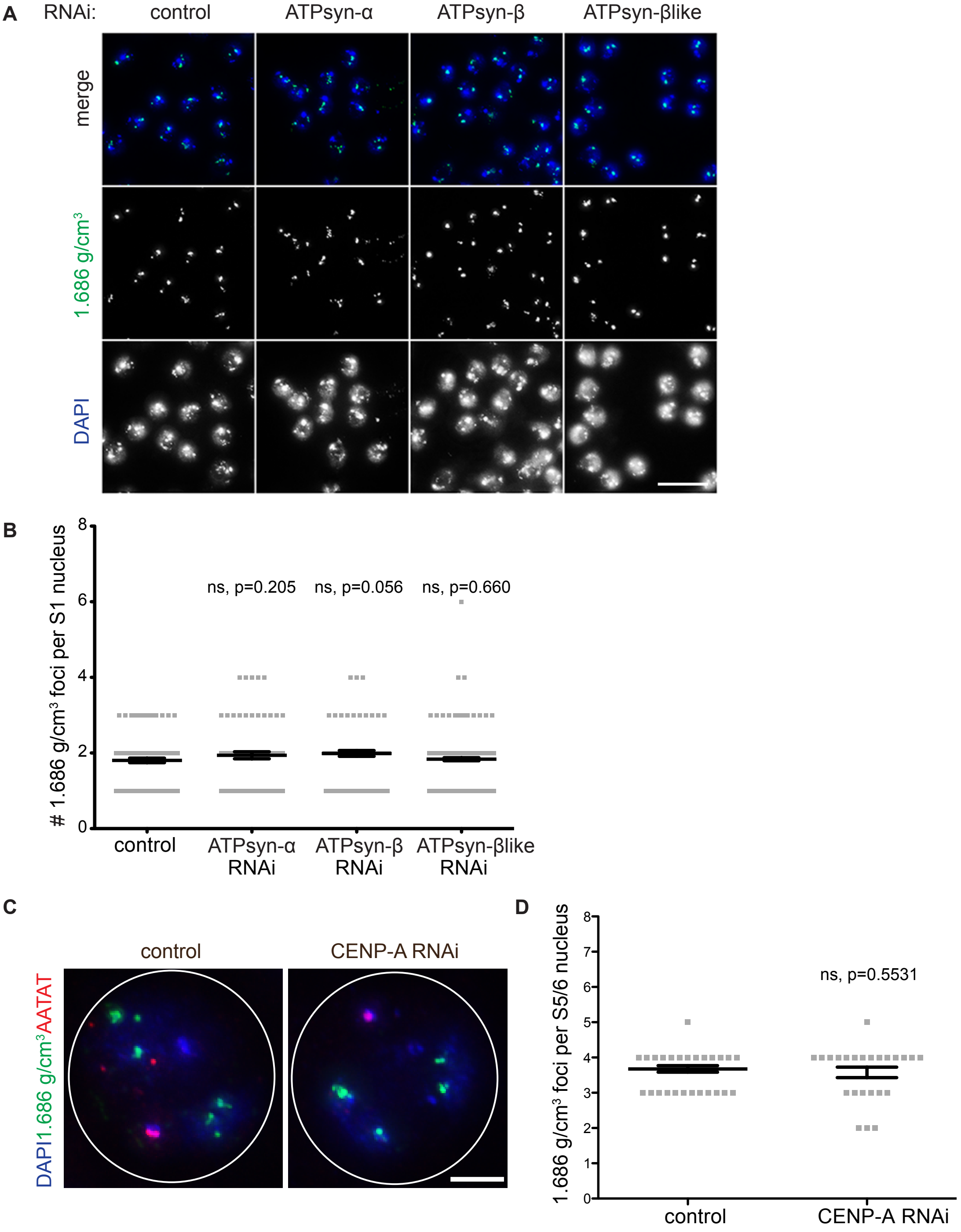

E

AATAT FISH pattern	control (%)	CENP-A RNAi (%)
1 spot	57.89	63.38
2 associated spots (<5μm)	31.57	28.16
2 unassociated spots (>5μm)	1.75	2.81
Diffuse spot	8.77	5.36

Supplementary Figure 3. FISH upon knockdown of ATPsyn-α/-β/-βlike or CENP-A.
A: Micrograph of 1.686 g/cm³ FISH (green) performed on control S1 nuclei or nuclei RNAi-depleted of ATPsyn-α, -β or -βlike (n=3). DNA is stained with DAPI (blue). Scale bar = 10 μm.
B: Quantitation of 1.686 g/cm³ foci in control S1 nuclei or nuclei RNAi-depleted of ATPsyn-α, -β or -βlike (data from one experiment, n=50 nuclei). Statistical significance was determined using an unpaired student's t-test. ns = p>0.05. Error bars = SEM.
C: Micrograph of 1.686 g/cm³ (green) and of AATAT (red) FISH performed on control S5/6 nuclei or nuclei RNAi-depleted of CENP-A (n=3). DNA is stained with DAPI; white circles outline the nucleus. Scale bar = 5 μm.
D: Quantitation of 1.686 g/cm³ foci in control S5/6 nuclei or nuclei RNAi-depleted of CENP-A (data from one experiment, n=25 nuclei).Statistical significance was determined using an unpaired student's t-test. ns = p>0.05. Error bars = SEM.
E: Quantitation (% nuclei, n=30) of the AATAT hybridisation pattern in control S5/6 nuclei or nuclei RNAi-depleted of CENP-A.

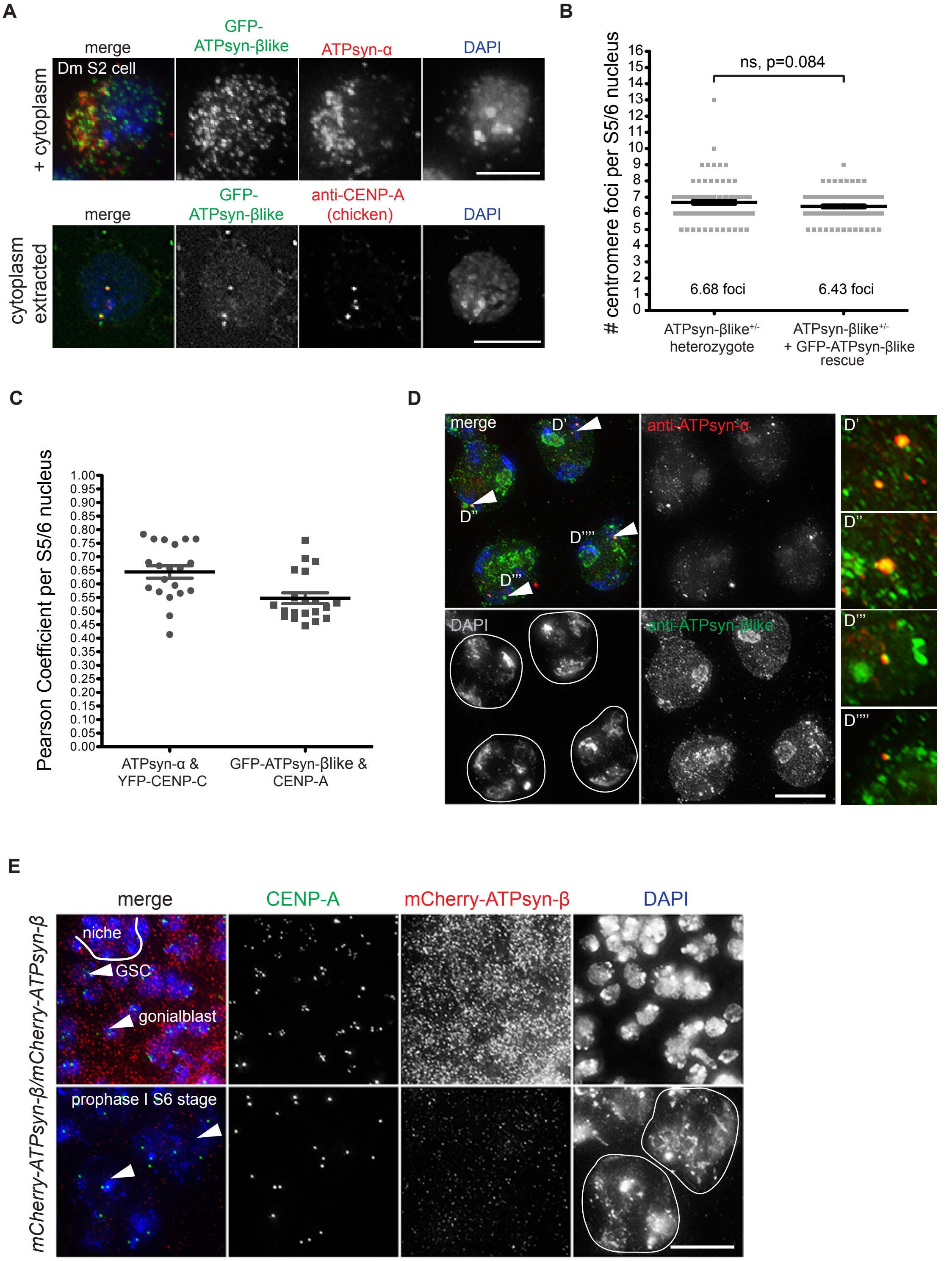

Supplementary Figure 4. ATPsyn-α and ATPsyn-βlike localisation at centromeres.

A: Top: Immuno-fluorescent micrograph of a transiently transfected *Dm* S2 cell expressing GFP-ATPsyn-βlike (green) stained with antibodies against ATPsyn-α (red) with cytoplasm intact (n=1). Bottom: Immunoflourescent micrograph of a transiently transfected *Dm* S2 cell expressing GFP-ATPsyn-βlike (green) stained with antibodies against ATPsyn-α (red) after extraction of the cytoplasm (n=3). DNA is stained with DAPI (blue). Scale bar = 5 μm.

B: Quantitation of the number of centromere foci per control S5/6 nuclei, or nuclei from flies lacking one functional copy of ATPsyn-βlike (ATPsyn-βlike$^{+/-}$ heterozygote, P element insertion) or nuclei from flies lacking one functional copy of ATPsyn-βlike expressing GFP-ATPsyn-βlike (rescue). Mean centromere foci values are indicated. Data is pooled from three individual experiments (n=100 nuclei). Statistical significance was determined using an unpaired student's t-test. ns = p>0.05. Error bars = SEM.

C: Pearson Coefficient per S5/6 nucleus for ATPsyn-α and YFP-CENP-C (0.6443 ± sd 0.1030, n=20 nuclei) and GFP-ATPsyn-βlike and CENP-A (0.5473 ± sd 0.0896, n=20 nuclei). Error bars = SEM. Scale bar = 5 μm.

D: Immuno-fluorescent micrograph of prophase I nuclei (S5/6 stage) stained with antibodies against ATPsyn-βlike (green) and ATPsyn-α (red) (n=3). DNA is stained with DAPI (blue). Inset panels D', D'', D''', D'''' and white arrowheads indicate overlapping foci (yellow) of ATPsyn-α and ATPsyn-βlike signals. Scale bar = 15 μm.

E: Immuno-fluorescent micrograph of adult testes expressing mCherry-ATPsyn-β (homozygous insertion) stained with antibodies against CENP-A (green) and RFP (red). DNA is stained with DAPI (blue). No colocalisation (n=2) was observed in germ cells indicated by white arrowheads; germ line stem cells (GSC) and gonialblast (top); prophase S5/6 nuclei (bottom). Scale bar = 10 μm.

Supplementary Figure 5

A

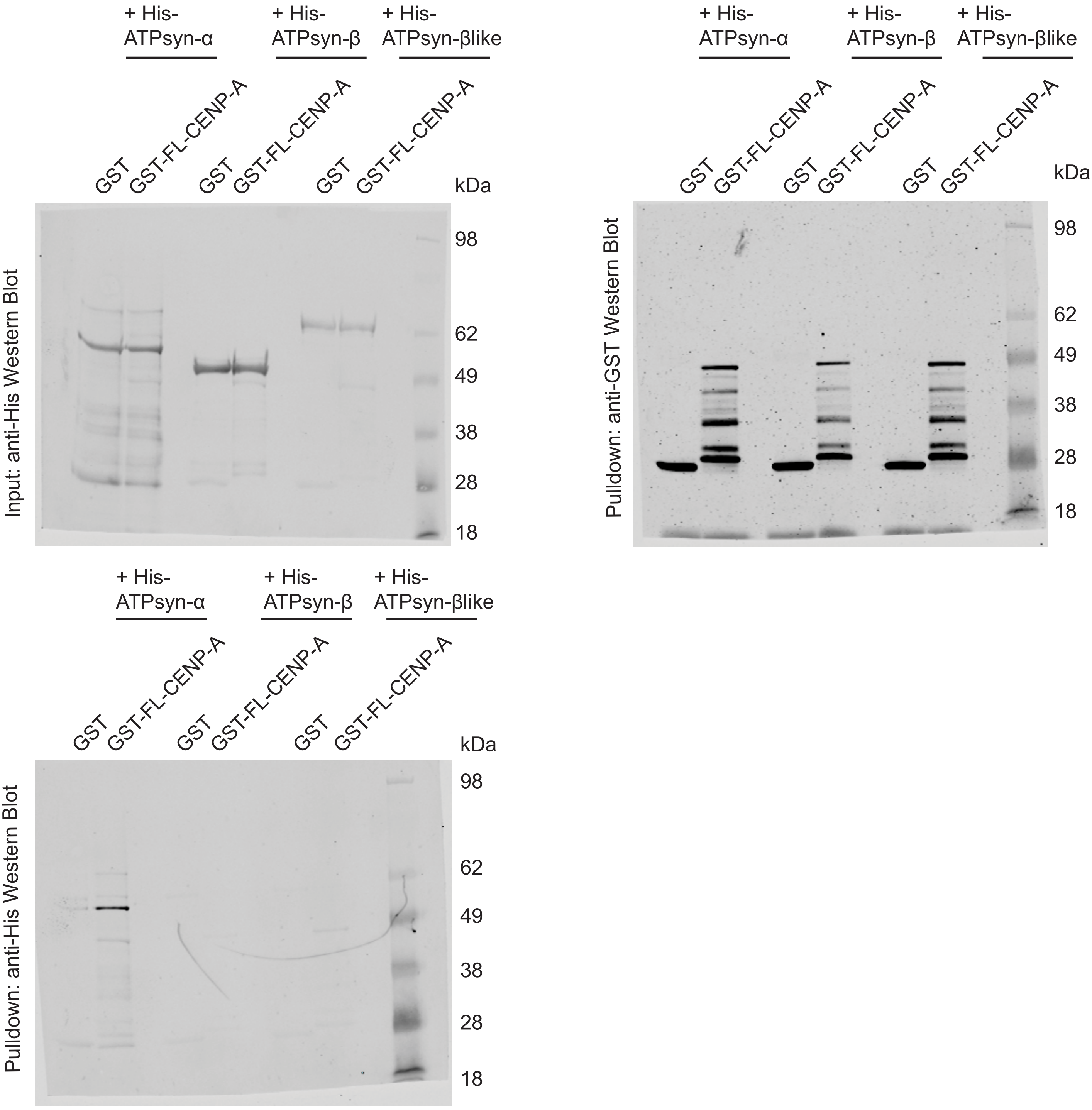

B

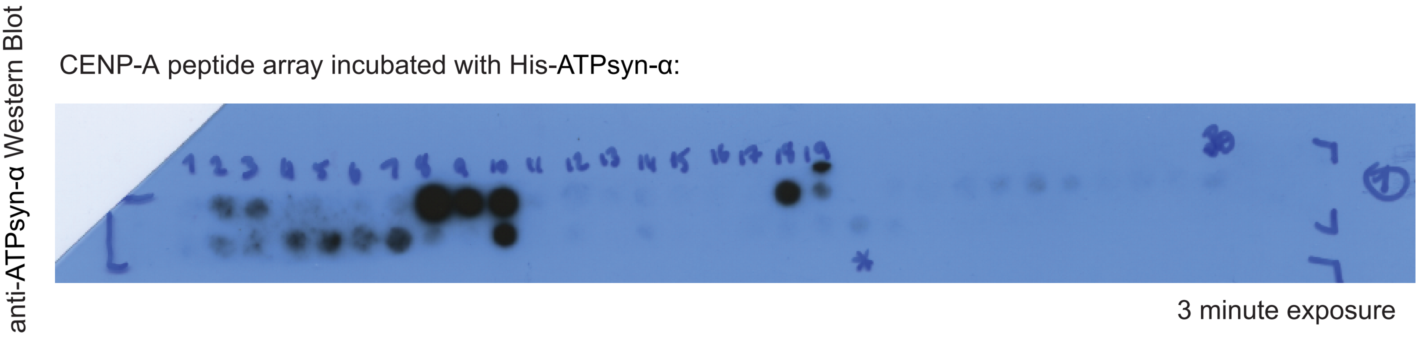

Supplementary Figure 5. Uncropped scans of western blots.
A: Related to Figure 4A. *In vitro* pulldown interaction with full-length GST-tagged CENP-A (GST-FL-CENP-A) or GST only and His-tagged ATPsyn-α, -β or -βlike revealed by western analysis with anti-His antibody (input, pulldown blots shown on left) or anti-GST antibody (pulldown blot shown on right).
B: Related to Figure 5A. CENP-A peptide array probed with recombinant His-ATPsyn-α, followed by western analysis with anti-ATPsyn-α antibody. Spots 1-19 encompass the CENP-A N terminus (amino acids 1-126).

Supplementary Table 1. List of RNAi lines used in this study.

Target Gene	Stock Number	Genotype
ATPsyn-α (*bellwether*)	BL28059	y[1] v[1]; P{y[+t7.7] v[+t1.8]=TRiP.JF02896}attP2
ATPsyn-α (*bellwether*)	V34664	w[1118]; P{GD11030}v34664
ATPsyn-β	BL27712	y[1] v[1]; P{y[+t7.7] v[+t1.8]=TRiP.JF02792}attP2
ATPsyn-β	V37812	w[1118] P{GD4967}v37812
ATPsyn-βlike	V22111	w[1118]; P{GD11634}v22111/TM3
ATPsyn-βlike	V106718	P{KK106259}VIE-260B
ATPsyn-γ	BL50543	y[1] v[1]; P{y[+t7.7] v[+t1.8]=TRiP.GLC01662}attP2
ATPsyn-γ	V16538	w[1118]; P{GD6339}v16538
ATPsyn-b	BL28062	y[1] v[1]; P{y[+t7.7] v[+t1.8]=TRiP.JF02899}attP2
ND-23	BL30487	y[1] sc[*] v[1]; P{y[+t7.7] v[+t1.8]=TRiP.HM05229}attP2
ND-51	BL29534	y[1] v[1]; P{y[+t7.7] v[+t1.8]=TRiP.HM05213}attP2
cid (*cenp-a*)	V43857	w[1118]; P{GD4436}v43857
cid (*cenp-a*)	V102090	P{KK110670}VIE-260B
Control (TRiP)	BL36303	y[1] v[1]; P{y[+t7.7]=CaryP}attP2
Control (VDRC, KK library)	V60100	w[1118]
Control (VDRC, GD library)	V60000	w[1118]
