# Supplementary material for: ATP synthase F1 subunits recruited to centromeres by CENP-A are required for male meiosis
Source: Nat Commun. 2018 Jul 13;9:2702. doi: 10.1038/s41467-018-05093-9 (PMC6045659; doi:10.1038/s41467-018-05093-9)
Supplement: Supplementary file 3 — Description of Additional Supplementary Information [file 41467_2018_5093_MOESM3_ESM.docx]

**Description of Additional Supplementary Files**

File Name: Supplementary Data 1

Description:

Table shows proteins identified in GST only control (columns labeled with A5 and A2) and GST-CENP-A N-terminal peptide (columns labeled with B5 and B2) pull down experiments. Peptides identified were searched against the Uniprot *Drosophila melanogaster* database and low confidence proteins were filtered to > 5 % false discovery rate (FDR). Proteins are identified by accession number (column A) and name (Column B). The data was analysed using Proteome Discoverer software (Thermo Scientific). Area (columns C & D) represents the relative level of the peptide in the sample; the score (columns E & I) represents the total confidence score for all identified peptides; coverage (columns F & J) represents the percentage of the total protein sequence identified by MS; # peptides (columns G & K) indicates the number of unique peptides identified and # PSM (columns H & L) indicates the number of peptide matches per protein including those redundantly identified. The protein length, molecular weight and theoretical isoelectric point are indicated in columns M-O.
